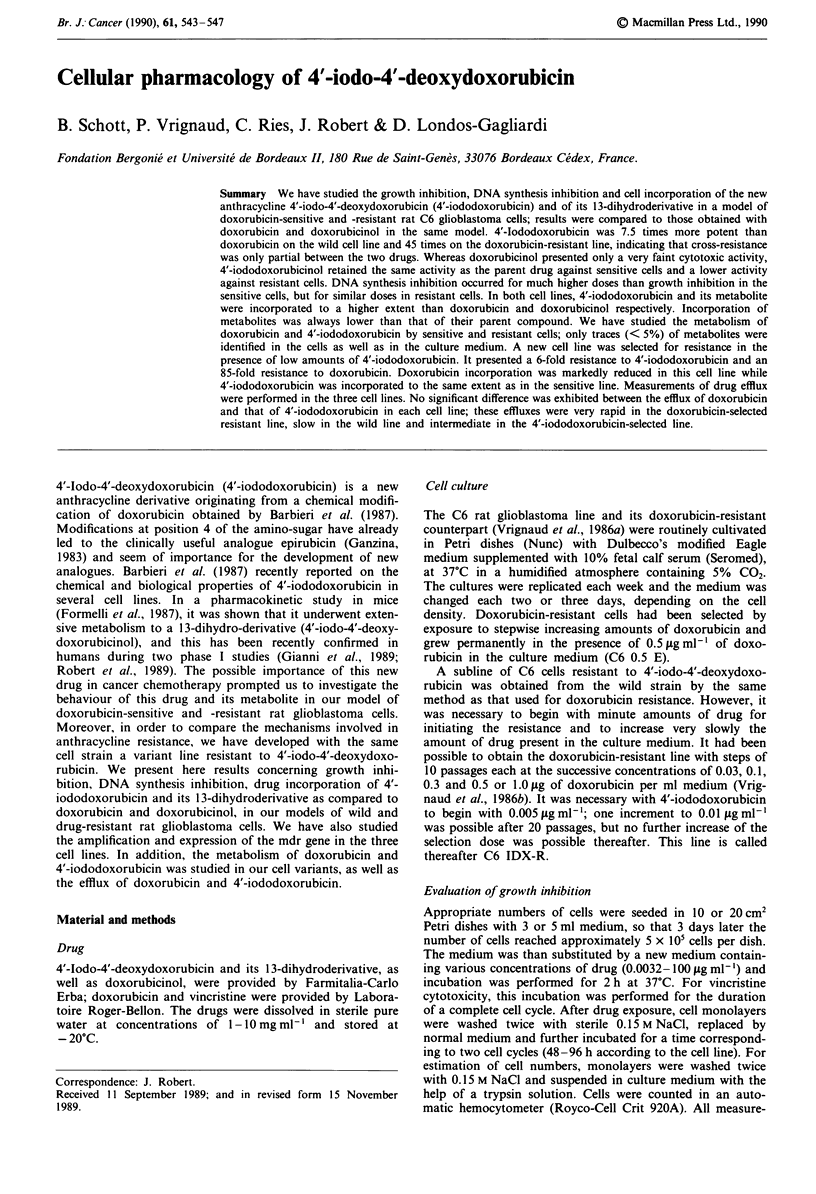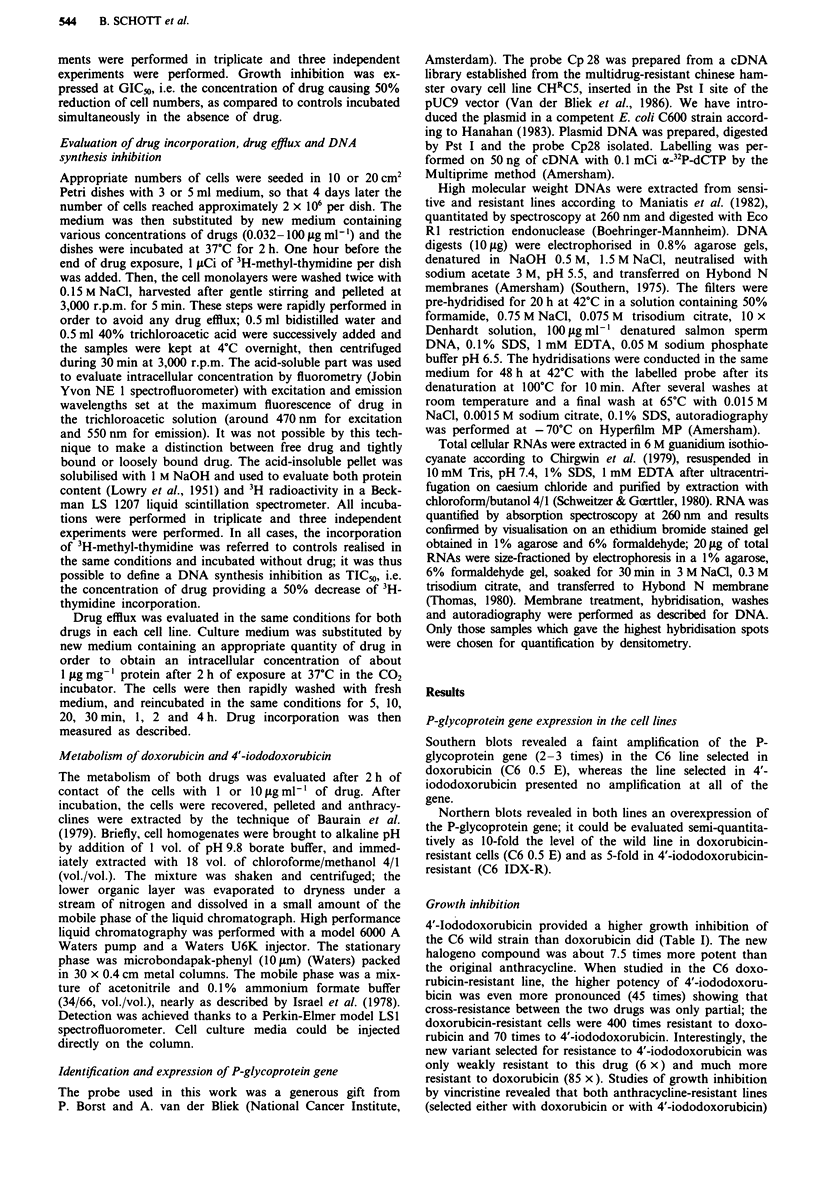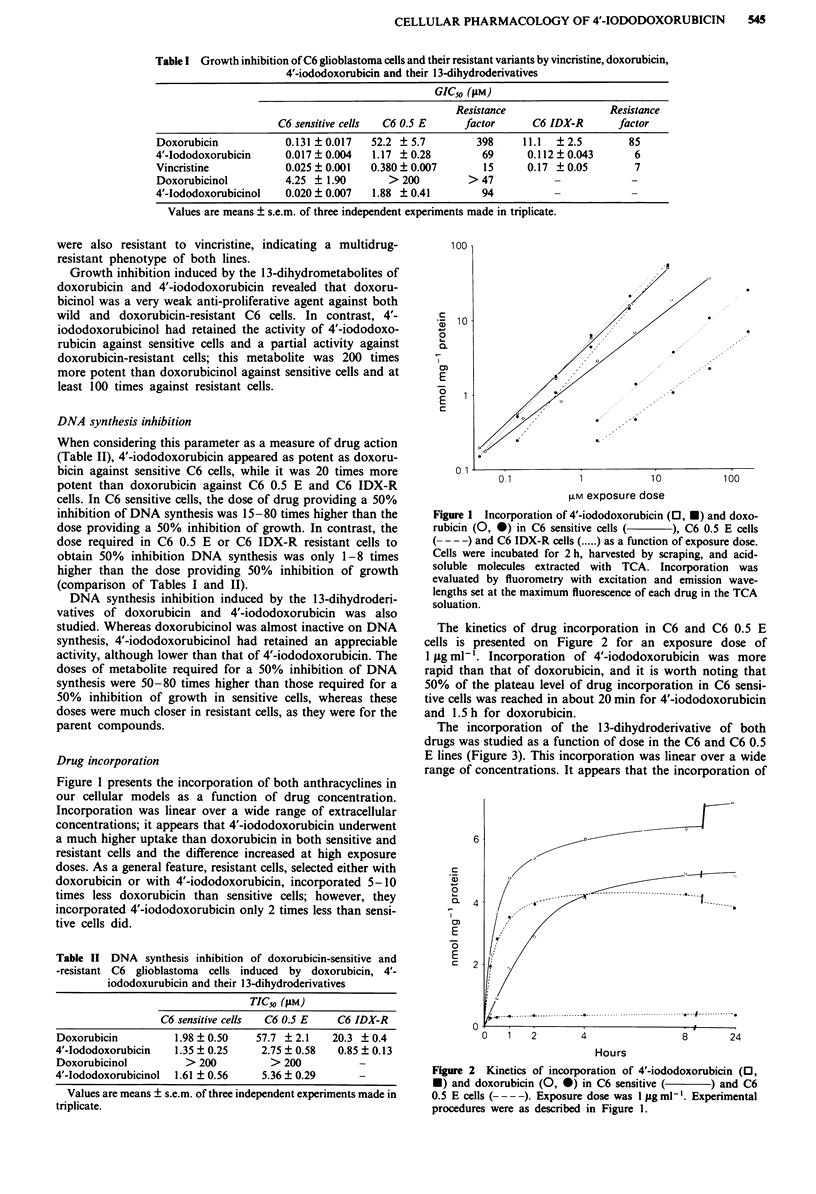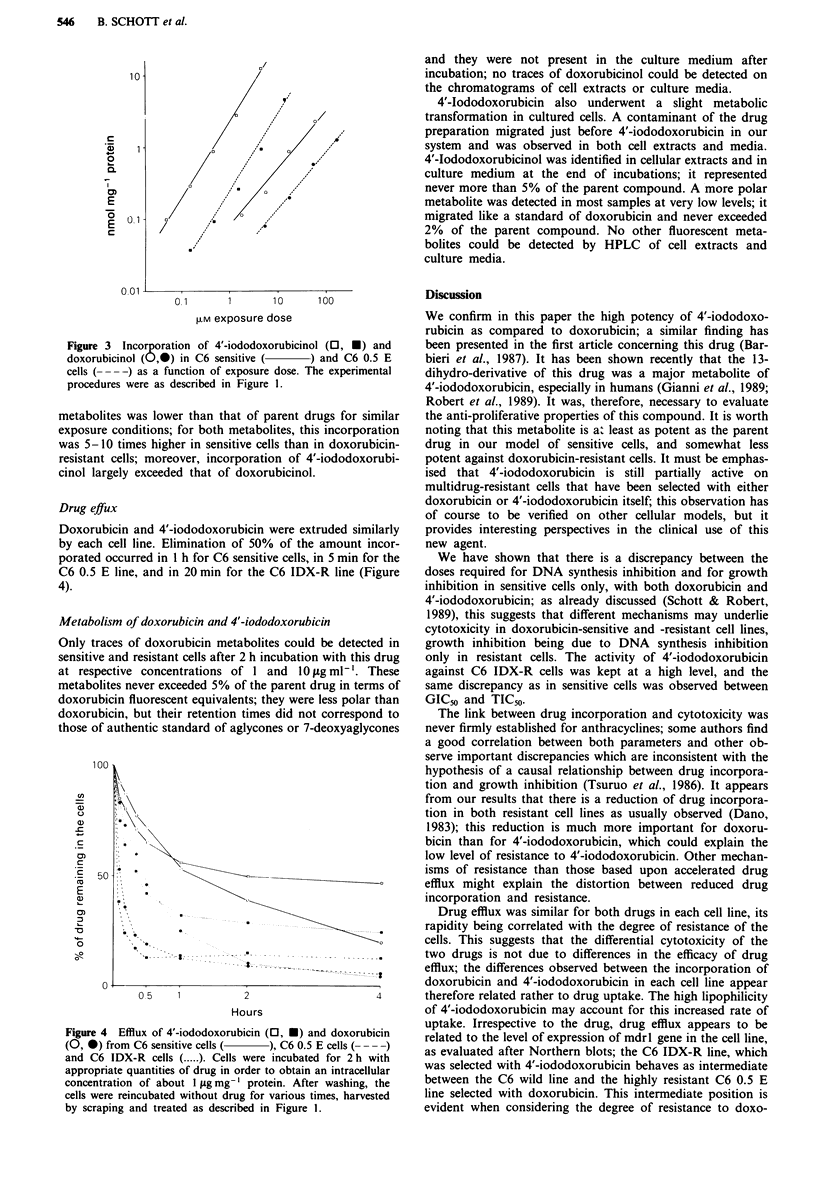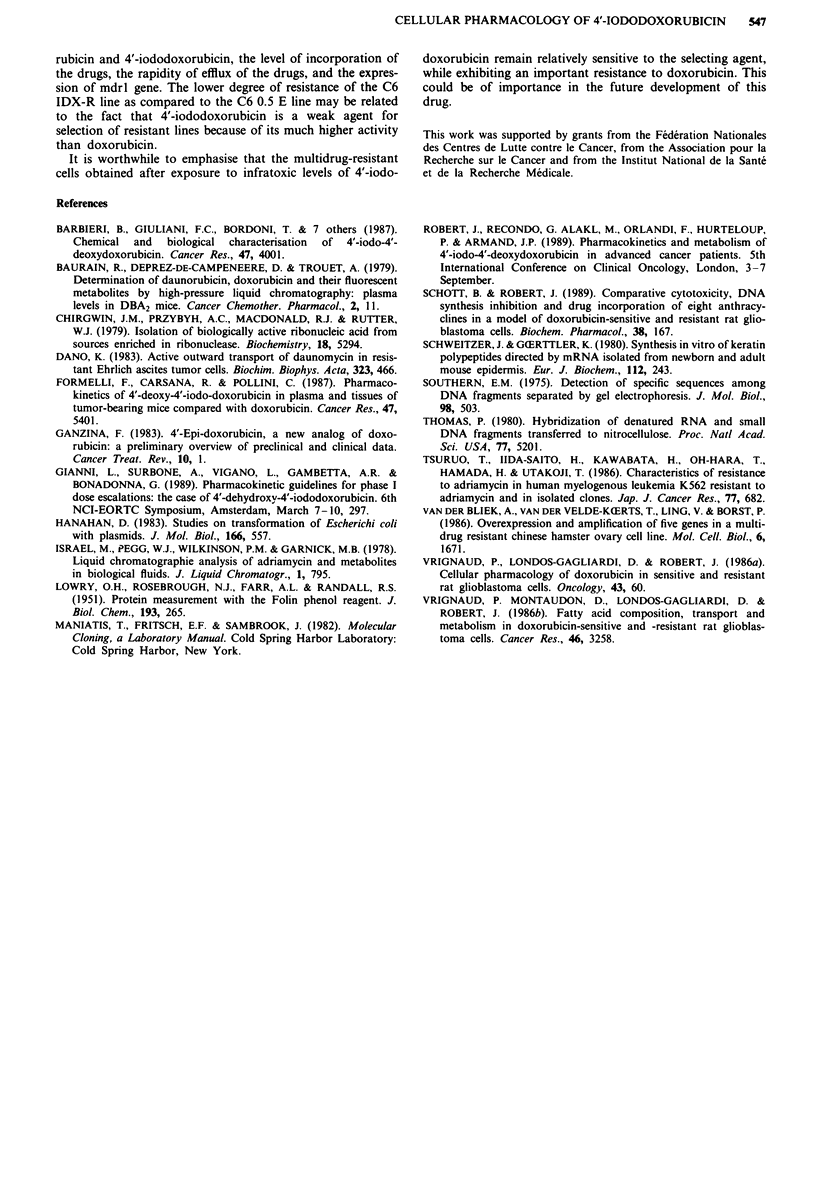# Cellular pharmacology of 4'-iodo-4'-deoxydoxorubicin.

**DOI:** 10.1038/bjc.1990.122

**Published:** 1990-04

**Authors:** B. Schott, P. Vrignaud, C. Ries, J. Robert, D. Londos-Gagliardi

**Affiliations:** Fondation Bergonié, Université de Bordeaux II, France.

## Abstract

We have studied the growth inhibition, DNA synthesis inhibition and cell incorporation of the new anthracycline 4'-iodo-4'-deoxydoxorubicin (4'-iododoxorubicin) and of its 13-dihydroderivative in a model of doxorubicin-sensitive and -resistant rat C6 glioblastoma cells; results were compared to those obtained with doxorubicin and doxorubicinol in the same model. 4'-Iododoxorubicin was 7.5 times more potent than doxorubicin on the wild cell line and 45 times on the doxorubicin-resistant line, indicating that cross-resistance was only partial between the two drugs. Whereas doxorubicinol presented only a very faint cytotoxic activity, 4'-iododoxorubicinol retained the same activity as the parent drug against sensitive cells and a lower activity against resistant cells. DNA synthesis inhibition occurred for much higher doses than growth inhibition in the sensitive cells, but for similar doses in resistant cells. In both cell lines, 4'-iododoxorubicin and its metabolite were incorporated to a higher extent than doxorubicin and doxorubicinol respectively. Incorporation of metabolites was always lower than that of their parent compound. We have studied the metabolism of doxorubicin and 4'-iododoxorubicin by sensitive and resistant cells; only traces (less than 5%) of metabolites were identified in the cells as well as in the culture medium. A new cell line was selected for resistance in the presence of low amounts of 4'-iododoxorubicin. It presented a 6-fold resistance to 4'-iododoxorubicin and an 85-fold resistance to doxorubicin. Doxorubicin incorporation was markedly reduced in this cell line while 4'-iododoxorubicin was incorporated to the same extent as in the sensitive line. Measurements of drug efflux were performed in the three cell lines. No significant difference was exhibited between the efflux of doxorubicin and that of 4'-iododoxorubicin in each cell line; these effluxes were very rapid in the doxorubicin-selected resistant line, slow in the wild line and intermediate in the 4'-iododoxorubicin-selected line.


Br. J. Cancer (1990), 61, 543–547 © Macmillan Press Ltd., 1990

### Cellular pharmacology of 4′-iodo-4′-deoxydoxorubicin

B. Schott, P. Vrignaud, C. Ries, J. Robert & D. Londos-Gagliardi

*Fondation Bergonié et Université de Bordeaux II, 180 Rue de Saint-Genès, 33076 Bordeaux Cédex, France.*

**Summary** We have studied the growth inhibition, DNA synthesis inhibition and cell incorporation of the new anthracycline 4′-iodo-4′-deoxydoxorubicin (4′-iododoxorubicin) and of its 13-dihydroderivative in a model of doxorubicin-sensitive and -resistant rat C6 glioblastoma cells; results were compared to those obtained with doxorubicin and doxorubicinol in the same model. 4′-Iododoxorubicin was 7.5 times more potent than doxorubicin on the wild cell line and 45 times on the doxorubicin-resistant line, indicating that cross-resistance was only partial between the two drugs. Whereas doxorubicinol presented only a very faint cytotoxic activity, 4′-iododoxorubicinol retained the same activity as the parent drug against sensitive cells and a lower activity against resistant cells. DNA synthesis inhibition occurred for much higher doses than growth inhibition in the sensitive cells, but for similar doses in resistant cells. In both cell lines, 4′-iododoxorubicin and its metabolite were incorporated to a higher extent than doxorubicin and doxorubicinol respectively. Incorporation of metabolites was always lower than that of their parent compound. We have studied the metabolism of doxorubicin and 4′-iododoxorubicin by sensitive and resistant cells; only traces (< 5%) of metabolites were identified in the cells as well as in the culture medium. A new cell line was selected for resistance in the presence of low amounts of 4′-iododoxorubicin. It presented a 6-fold resistance to 4′-iododoxorubicin and an 85-fold resistance to doxorubicin. Doxorubicin incorporation was markedly reduced in this cell line while 4′-iododoxorubicin was incorporated to the same extent as in the sensitive line. Measurements of drug efflux were performed in the three cell lines. No significant difference was exhibited between the efflux of doxorubicin and that of 4′-iododoxorubicin in each cell line; these effluxes were very rapid in the doxorubicin-selected resistant line, slow in the wild line and intermediate in the 4′-iododoxorubicin-selected line.

4′-Iodo-4′-deoxydoxorubicin (4′-iododoxorubicin) is a new anthracycline derivative originating from a chemical modification of doxorubicin obtained by Barbieri *et al.* (1987). Modifications at position 4 of the amino-sugar have already led to the clinically useful analogue epirubicin (Ganzina, 1983) and seem of importance for the development of new analogues. Barbieri *et al.* (1987) recently reported on the chemical and biological properties of 4′-iododoxorubicin in several cell lines. In a pharmacokinetic study in mice (Formelli *et al.*, 1987), it was shown that it underwent extensive metabolism to a 13-dihydro-derivative (4′-iodo-4′-deoxydoxorubicinol), and this has been recently confirmed in humans during two phase I studies (Gianni *et al.*, 1989; Robert *et al.*, 1989). The possible importance of this new drug in cancer chemotherapy prompted us to investigate the behaviour of this drug and its metabolite in our model of doxorubicin-sensitive and -resistant rat glioblastoma cells. Moreover, in order to compare the mechanisms involved in anthracycline resistance, we have developed with the same cell strain a variant line resistant to 4′-iodo-4′-deoxydoxorubicin. We present here results concerning growth inhibition, DNA synthesis inhibition, drug incorporation of 4′-iododoxorubicin and its 13-dihydroderivative as compared to doxorubicin and doxorubicinol, in our models of wild and drug-resistant rat glioblastoma cells. We have also studied the amplification and expression of the mdr gene in the three cell lines. In addition, the metabolism of doxorubicin and 4′-iododoxorubicin was studied in our cell variants, as well as the efflux of doxorubicin and 4′-iododoxorubicin.

### Material and methods

### *Drug*

4′-Iodo-4′-deoxydoxorubicin and its 13-dihydroderivative, as well as doxorubicinol, were provided by Farmitalia-Carlo Erba; doxorubicin and vincristine were provided by Laboratoire Roger-Bellon. The drugs were dissolved in sterile pure water at concentrations of $1-10\,mg\,ml^{-1}$ and stored at $-20°C$.

Correspondence: J. Robert.
Received 11 September 1989; and in revised form 15 November 1989.

### *Cell culture*

The C6 rat glioblastoma line and its doxorubicin-resistant counterpart (Vrignaud *et al.*, 1986*a*) were routinely cultivated in Petri dishes (Nunc) with Dulbecco's modified Eagle medium supplemented with 10% fetal calf serum (Seromed), at 37°C in a humidified atmosphere containing 5% $CO_2$. The cultures were replicated each week and the medium was changed each two or three days, depending on the cell density. Doxorubicin-resistant cells had been selected by exposure to stepwise increasing amounts of doxorubicin and grew permanently in the presence of $0.5\,\mu g\,ml^{-1}$ of doxorubicin in the culture medium (C6 0.5 E).

A subline of C6 cells resistant to 4′-iodo-4′-deoxydoxorubicin was obtained from the wild strain by the same method as that used for doxorubicin resistance. However, it was necessary to begin with minute amounts of drug for initiating the resistance and to increase very slowly the amount of drug present in the culture medium. It had been possible to obtain the doxorubicin-resistant line with steps of 10 passages each at the successive concentrations of 0.03, 0.1, 0.3 and 0.5 or 1.0 µg of doxorubicin per ml medium (Vrignaud *et al.*, 1986*b*). It was necessary with 4′-iododoxorubicin to begin with $0.005\,\mu g\,ml^{-1}$; one increment to $0.01\,\mu g\,ml^{-1}$ was possible after 20 passages, but no further increase of the selection dose was possible thereafter. This line is called thereafter C6 IDX-R.

### *Evaluation of growth inhibition*

Appropriate numbers of cells were seeded in 10 or $20\,cm^2$ Petri dishes with 3 or 5 ml medium, so that 3 days later the number of cells reached approximately $5 \times 10^5$ cells per dish. The medium was than substituted by a new medium containing various concentrations of drug ($0.0032-100\,\mu g\,ml^{-1}$) and incubation was performed for 2 h at 37°C. For vincristine cytotoxicity, this incubation was performed for the duration of a complete cell cycle. After drug exposure, cell monolayers were washed twice with sterile 0.15 M NaCl, replaced by normal medium and further incubated for a time corresponding to two cell cycles (48–96 h according to the cell line). For estimation of cell numbers, monolayers were washed twice with 0.15 M NaCl and suspended in culture medium with the help of a trypsin solution. Cells were counted in an automatic hemocytometer (Royco-Cell Crit 920A). All measurements were performed in triplicate and three independent experiments were performed. Growth inhibition was expressed at $GIC_{50}$, i.e. the concentration of drug causing 50% reduction of cell numbers, as compared to controls incubated simultaneously in the absence of drug.

*Evaluation of drug incorporation, drug efflux and DNA synthesis inhibition*

Appropriate numbers of cells were seeded in 10 or 20 $cm^2$ Petri dishes with 3 or 5 ml medium, so that 4 days later the number of cells reached approximately $2 \times 10^6$ per dish. The medium was then substituted by new medium containing various concentrations of drugs (0.032–100 $\mu g\ ml^{-1}$) and the dishes were incubated at 37°C for 2 h. One hour before the end of drug exposure, 1 $\mu$Ci of $^3$H-methyl-thymidine per dish was added. Then, the cell monolayers were washed twice with 0.15 M NaCl, harvested after gentle stirring and pelleted at 3,000 r.p.m. for 5 min. These steps were rapidly performed in order to avoid any drug efflux; 0.5 ml bidistilled water and 0.5 ml 40% trichloroacetic acid were successively added and the samples were kept at 4°C overnight, then centrifuged during 30 min at 3,000 r.p.m. The acid-soluble part was used to evaluate intracellular concentration by fluorometry (Jobin Yvon NE 1 spectrofluorometer) with excitation and emission wavelengths set at the maximum fluorescence of drug in the trichloroacetic solution (around 470 nm for excitation and 550 nm for emission). It was not possible by this technique to make a distinction between free drug and tightly bound or loosely bound drug. The acid-insoluble pellet was solubilised with 1 M NaOH and used to evaluate both protein content (Lowry *et al.*, 1951) and $^3$H radioactivity in a Beckman LS 1207 liquid scintillation spectrometer. All incubations were performed in triplicate and three independent experiments were performed. In all cases, the incorporation of $^3$H-methyl-thymidine was referred to controls realised in the same conditions and incubated without drug; it was thus possible to define a DNA synthesis inhibition as $TIC_{50}$, i.e. the concentration of drug providing a 50% decrease of $^3$H-thymidine incorporation.

Drug efflux was evaluated in the same conditions for both drugs in each cell line. Culture medium was substituted by new medium containing an appropriate quantity of drug in order to obtain an intracellular concentration of about 1 $\mu g\ mg^{-1}$ protein after 2 h of exposure at 37°C in the $CO_2$ incubator. The cells were then rapidly washed with fresh medium, and reincubated in the same conditions for 5, 10, 20, 30 min, 1, 2 and 4 h. Drug incorporation was then measured as described.

*Metabolism of doxorubicin and 4'-iododoxorubicin*

The metabolism of both drugs was evaluated after 2 h of contact of the cells with 1 or 10 $\mu g\ ml^{-1}$ of drug. After incubation, the cells were recovered, pelleted and anthracyclines were extracted by the technique of Baurain *et al.* (1979). Briefly, cell homogenates were brought to alkaline pH by addition of 1 vol. of pH 9.8 borate buffer, and immediately extracted with 18 vol. of chloroforme/methanol 4/1 (vol./vol.). The mixture was shaken and centrifuged; the lower organic layer was evaporated to dryness under a stream of nitrogen and dissolved in a small amount of the mobile phase of the liquid chromatograph. High performance liquid chromatography was performed with a model 6000 A Waters pump and a Waters U6K injector. The stationary phase was microbondapak-phenyl (10 $\mu$m) (Waters) packed in 30 × 0.4 cm metal columns. The mobile phase was a mixture of acetonitrile and 0.1% ammonium formate buffer (34/66, vol./vol.), nearly as described by Israel *et al.* (1978). Detection was achieved thanks to a Perkin-Elmer model LS1 spectrofluorometer. Cell culture media could be injected directly on the column.

*Identification and expression of P-glycoprotein gene*

The probe used in this work was a generous gift from P. Borst and A. van der Bliek (National Cancer Institute, Amsterdam). The probe Cp 28 was prepared from a cDNA library established from the multidrug-resistant chinese hamster ovary cell line $CH^RC5$, inserted in the Pst I site of the pUC9 vector (Van der Bliek *et al.*, 1986). We have introduced the plasmid in a competent *E. coli* C600 strain according to Hanahan (1983). Plasmid DNA was prepared, digested by Pst I and the probe Cp28 isolated. Labelling was performed on 50 ng of cDNA with 0.1 mCi $\alpha$-$^{32}$P-dCTP by the Multiprime method (Amersham).

High molecular weight DNAs were extracted from sensitive and resistant lines according to Maniatis *et al.* (1982), quantitated by spectroscopy at 260 nm and digested with Eco R1 restriction endonuclease (Boehringer-Mannheim). DNA digests (10 $\mu$g) were electrophorised in 0.8% agarose gels, denatured in NaOH 0.5 M, 1.5 M NaCl, neutralised with sodium acetate 3 M, pH 5.5, and transferred on Hybond N membranes (Amersham) (Southern, 1975). The filters were pre-hydridised for 20 h at 42°C in a solution containing 50% formamide, 0.75 M NaCl, 0.075 M trisodium citrate, 10 × Denhardt solution, 100 $\mu g\ ml^{-1}$ denatured salmon sperm DNA, 0.1% SDS, 1 mM EDTA, 0.05 M sodium phosphate buffer pH 6.5. The hydridisations were conducted in the same medium for 48 h at 42°C with the labelled probe after its denaturation at 100°C for 10 min. After several washes at room temperature and a final wash at 65°C with 0.015 M NaCl, 0.0015 M sodium citrate, 0.1% SDS, autoradiography was performed at −70°C on Hyperfilm MP (Amersham).

Total cellular RNAs were extracted in 6 M guanidium isothiocyanate according to Chirgwin *et al.* (1979), resuspended in 10 mM Tris, pH 7.4, 1% SDS, 1 mM EDTA after ultracentrifugation on caesium chloride and purified by extraction with chloroform/butanol 4/1 (Schweitzer & Gœrttler, 1980). RNA was quantified by absorption spectroscopy at 260 nm and results confirmed by visualisation on an ethidium bromide stained gel obtained in 1% agarose and 6% formaldehyde; 20 $\mu$g of total RNAs were size-fractioned by electrophoresis in a 1% agarose, 6% formaldehyde gel, soaked for 30 min in 3 M NaCl, 0.3 M trisodium citrate, and transferred to Hybond N membrane (Thomas, 1980). Membrane treatment, hybridisation, washes and autoradiography were performed as described for DNA. Only those samples which gave the highest hybridisation spots were chosen for quantification by densitometry.

### Results

*P-glycoprotein gene expression in the cell lines*

Southern blots revealed a faint amplification of the P-glycoprotein gene (2–3 times) in the C6 line selected in doxorubicin (C6 0.5 E), whereas the line selected in 4'-iododoxorubicin presented no amplification at all of the gene.

Northern blots revealed in both lines an overexpression of the P-glycoprotein gene; it could be evaluated semi-quantitatively as 10-fold the level of the wild line in doxorubicin-resistant cells (C6 0.5 E) and as 5-fold in 4'-iododoxorubicin-resistant (C6 IDX-R).

*Growth inhibition*

4'-Iododoxorubicin provided a higher growth inhibition of the C6 wild strain than doxorubicin did (Table I). The new halogeno compound was about 7.5 times more potent than the original anthracycline. When studied in the C6 doxorubicin-resistant line, the higher potency of 4'-iododoxorubicin was even more pronounced (45 times) showing that cross-resistance between the two drugs was only partial; the doxorubicin-resistant cells were 400 times resistant to doxorubicin and 70 times to 4'-iododoxorubicin. Interestingly, the new variant selected for resistance to 4'-iododoxorubicin was only weakly resistant to this drug (6 ×) and much more resistant to doxorubicin (85 ×). Studies of growth inhibition by vincristine revealed that both anthracycline-resistant lines (selected either with doxorubicin or with 4'-iododoxorubicin)

**Table I** Growth inhibition of C6 glioblastoma cells and their resistant variants by vincristine, doxorubicin, 4'-iododoxorubicin and their 13-dihydroderivatives

| | *$GIC_{50}$ (μM)* | | | | |
|---|---|---|---|---|---|
| | *C6 sensitive cells* | *C6 0.5 E* | *Resistance factor* | *C6 IDX-R* | *Resistance factor* |
| Doxorubicin | 0.131 ± 0.017 | 52.2 ± 5.7 | 398 | 11.1 ± 2.5 | 85 |
| 4'-Iododoxorubicin | 0.017 ± 0.004 | 1.17 ± 0.28 | 69 | 0.112 ± 0.043 | 6 |
| Vincristine | 0.025 ± 0.001 | 0.380 ± 0.007 | 15 | 0.17 ± 0.05 | 7 |
| Doxorubicinol | 4.25 ± 1.90 | > 200 | > 47 | – | – |
| 4'-Iododoxorubicinol | 0.020 ± 0.007 | 1.88 ± 0.41 | 94 | – | – |

Values are means ± s.e.m. of three independent experiments made in triplicate.

were also resistant to vincristine, indicating a multidrug-resistant phenotype of both lines.

Growth inhibition induced by the 13-dihydrometabolites of doxorubicin and 4'-iododoxorubicin revealed that doxorubicinol was a very weak anti-proliferative agent against both wild and doxorubicin-resistant C6 cells. In contrast, 4'-iododoxorubicinol had retained the activity of 4'-iododoxorubicin against sensitive cells and a partial activity against doxorubicin-resistant cells; this metabolite was 200 times more potent than doxorubicinol against sensitive cells and at least 100 times against resistant cells.

*DNA synthesis inhibition*

When considering this parameter as a measure of drug action (Table II), 4'-iododoxorubicin appeared as potent as doxorubicin against sensitive C6 cells, while it was 20 times more potent than doxorubicin against C6 0.5 E and C6 IDX-R cells. In C6 sensitive cells, the dose of drug providing a 50% inhibition of DNA synthesis was 15–80 times higher than the dose providing a 50% inhibition of growth. In contrast, the dose required in C6 0.5 E or C6 IDX-R resistant cells to obtain 50% inhibition DNA synthesis was only 1–8 times higher than the dose providing 50% inhibition of growth (comparison of Tables I and II).

DNA synthesis inhibition induced by the 13-dihydroderivatives of doxorubicin and 4'-iododoxorubicin was also studied. Whereas doxorubicinol was almost inactive on DNA synthesis, 4'-iododoxorubicinol had retained an appreciable activity, although lower than that of 4'-iododoxorubicin. The doses of metabolite required for a 50% inhibition of DNA synthesis were 50–80 times higher than those required for a 50% inhibition of growth in sensitive cells, whereas these doses were much closer in resistant cells, as they were for the parent compounds.

*Drug incorporation*

Figure 1 presents the incorporation of both anthracyclines in our cellular models as a function of drug concentration. Incorporation was linear over a wide range of extracellular concentrations; it appears that 4'-iododoxorubicin underwent a much higher uptake than doxorubicin in both sensitive and resistant cells and the difference increased at high exposure doses. As a general feature, resistant cells, selected either with doxorubicin or with 4'-iododoxorubicin, incorporated 5–10 times less doxorubicin than sensitive cells; however, they incorporated 4'-iododoxorubicin only 2 times less than sensitive cells did.

**Table II** DNA synthesis inhibition of doxorubicin-sensitive and -resistant C6 glioblastoma cells induced by doxorubicin, 4'-iododoxurubicin and their 13-dihydroderivatives

| | *$TIC_{50}$ (μM)* | | |
|---|---|---|---|
| | *C6 sensitive cells* | *C6 0.5 E* | *C6 IDX-R* |
| Doxorubicin | 1.98 ± 0.50 | 57.7 ± 2.1 | 20.3 ± 0.4 |
| 4'-Iododoxorubicin | 1.35 ± 0.25 | 2.75 ± 0.58 | 0.85 ± 0.13 |
| Doxorubicinol | > 200 | > 200 | – |
| 4'-Iododoxorubicinol | 1.61 ± 0.56 | 5.36 ± 0.29 | – |

Values are means ± s.e.m. of three independent experiments made in triplicate.

**Figure 1** Incorporation of 4'-iododoxorubicin (□, ■) and doxorubicin (O, ●) in C6 sensitive cells (———), C6 0.5 E cells (- - - -) and C6 IDX-R cells (.....) as a function of exposure dose. Cells were incubated for 2 h, harvested by scraping, and acid-soluble molecules extracted with TCA. Incorporation was evaluated by fluorometry with excitation and emission wavelengths set at the maximum fluorescence of each drug in the TCA soluation.

The kinetics of drug incorporation in C6 and C6 0.5 E cells is presented on Figure 2 for an exposure dose of 1 $\mu g\ ml^{-1}$. Incorporation of 4'-iododoxorubicin was more rapid than that of doxorubicin, and it is worth noting that 50% of the plateau level of drug incorporation in C6 sensitive cells was reached in about 20 min for 4'-iododoxorubicin and 1.5 h for doxorubicin.

The incorporation of the 13-dihydroderivative of both drugs was studied as a function of dose in the C6 and C6 0.5 E lines (Figure 3). This incorporation was linear over a wide range of concentrations. It appears that the incorporation of

**Figure 2** Kinetics of incorporation of 4'-iododoxorubicin (□, ■) and doxorubicin (O, ●) in C6 sensitive (———) and C6 0.5 E cells (- - - -). Exposure dose was 1 $\mu g\ ml^{-1}$. Experimental procedures were as described in Figure 1.

**Figure 3** Incorporation of 4′-iododoxorubicinol (□, ■) and doxorubicinol (○,●) in C6 sensitive (————) and C6 0.5 E cells (- - - -) as a function of exposure dose. The experimental procedures were as described in Figure 1.

metabolites was lower than that of parent drugs for similar exposure conditions; for both metabolites, this incorporation was 5–10 times higher in sensitive cells than in doxorubicin-resistant cells; moreover, incorporation of 4′-iododoxorubicinol largely exceeded that of doxorubicinol.

*Drug effux*

Doxorubicin and 4′-iododoxorubicin were extruded similarly by each cell line. Elimination of 50% of the amount incorporated occurred in 1 h for C6 sensitive cells, in 5 min for the C6 0.5 E line, and in 20 min for the C6 IDX-R line (Figure 4).

*Metabolism of doxorubicin and 4′-iododoxorubicin*

Only traces of doxorubicin metabolites could be detected in sensitive and resistant cells after 2 h incubation with this drug at respective concentrations of 1 and 10 μg ml$^{-1}$. These metabolites never exceeded 5% of the parent drug in terms of doxorubicin fluorescent equivalents; they were less polar than doxorubicin, but their retention times did not correspond to those of authentic standard of aglycones or 7-deoxyaglycones and they were not present in the culture medium after incubation; no traces of doxorubicinol could be detected on the chromatograms of cell extracts or culture media.

**Figure 4** Efflux of 4′-iododoxorubicin (□, ■) and doxorubicin (○, ●) from C6 sensitive cells (————), C6 0.5 E cells (- - - -) and C6 IDX-R cells (.....). Cells were incubated for 2 h with appropriate quantities of drug in order to obtain an intracellular concentration of about 1 μg mg$^{-1}$ protein. After washing, the cells were reincubated without drug for various times, harvested by scraping and treated as described in Figure 1.

4′-Iododoxorubicin also underwent a slight metabolic transformation in cultured cells. A contaminant of the drug preparation migrated just before 4′-iododoxorubicin in our system and was observed in both cell extracts and media. 4′-Iododoxorubicinol was identified in cellular extracts and in culture medium at the end of incubations; it represented never more than 5% of the parent compound. A more polar metabolite was detected in most samples at very low levels; it migrated like a standard of doxorubicin and never exceeded 2% of the parent compound. No other fluorescent metabolites could be detected by HPLC of cell extracts and culture media.

### Discussion

We confirm in this paper the high potency of 4′-iododoxorubicin as compared to doxorubicin; a similar finding has been presented in the first article concerning this drug (Barbieri *et al.*, 1987). It has been shown recently that the 13-dihydro-derivative of this drug was a major metabolite of 4′-iododoxorubicin, especially in humans (Gianni *et al.*, 1989; Robert *et al.*, 1989). It was, therefore, necessary to evaluate the anti-proliferative properties of this compound. It is worth noting that this metabolite is at least as potent as the parent drug in our model of sensitive cells, and somewhat less potent against doxorubicin-resistant cells. It must be emphasised that 4′-iododoxorubicin is still partially active on multidrug-resistant cells that have been selected with either doxorubicin or 4′-iododoxorubicin itself; this observation has of course to be verified on other cellular models, but it provides interesting perspectives in the clinical use of this new agent.

We have shown that there is a discrepancy between the doses required for DNA synthesis inhibition and for growth inhibition in sensitive cells only, with both doxorubicin and 4′-iododoxorubicin; as already discussed (Schott & Robert, 1989), this suggests that different mechanisms may underlie cytotoxicity in doxorubicin-sensitive and -resistant cell lines, growth inhibition being due to DNA synthesis inhibition only in resistant cells. The activity of 4′-iododoxorubicin against C6 IDX-R cells was kept at a high level, and the same discrepancy as in sensitive cells was observed between $GIC_{50}$ and $TIC_{50}$.

The link between drug incorporation and cytotoxicity was never firmly established for anthracyclines; some authors find a good correlation between both parameters and other observe important discrepancies which are inconsistent with the hypothesis of a causal relationship between drug incorporation and growth inhibition (Tsuruo *et al.*, 1986). It appears from our results that there is a reduction of drug incorporation in both resistant cell lines as usually observed (Dano, 1983); this reduction is much more important for doxorubicin than for 4′-iododoxorubicin, which could explain the low level of resistance to 4′-iododoxorubicin. Other mechanisms of resistance than those based upon accelerated drug efflux might explain the distortion between reduced drug incorporation and resistance.

Drug efflux was similar for both drugs in each cell line, its rapidity being correlated with the degree of resistance of the cells. This suggests that the differential cytotoxicity of the two drugs is not due to differences in the efficacy of drug efflux; the differences observed between the incorporation of doxorubicin and 4′-iododoxorubicin in each cell line appear therefore related rather to drug uptake. The high lipophilicity of 4′-iododoxorubicin may account for this increased rate of uptake. Irrespective to the drug, drug efflux appears to be related to the level of expression of mdr1 gene in the cell line, as evaluated after Northern blots; the C6 IDX-R line, which was selected with 4′-iododoxorubicin behaves as intermediate between the C6 wild line and the highly resistant C6 0.5 E line selected with doxorubicin. This intermediate position is evident when considering the degree of resistance to doxorubicin and 4'-iododoxorubicin, the level of incorporation of the drugs, the rapidity of efflux of the drugs, and the expression of mdr1 gene. The lower degree of resistance of the C6 IDX-R line as compared to the C6 0.5 E line may be related to the fact that 4'-iododoxorubicin is a weak agent for selection of resistant lines because of its much higher activity than doxorubicin.

It is worthwhile to emphasise that the multidrug-resistant cells obtained after exposure to infratoxic levels of 4'-iododoxorubicin remain relatively sensitive to the selecting agent, while exhibiting an important resistance to doxorubicin. This could be of importance in the future development of this drug.

This work was supported by grants from the Fédération Nationales des Centres de Lutte contre le Cancer, from the Association pour la Recherche sur le Cancer and from the Institut National de la Santé et de la Recherche Médicale.

**References**

BARBIERI, B., GIULIANI, F.C., BORDONI, T. & 7 others (1987). Chemical and biological characterisation of 4'-iodo-4'-deoxydoxorubicin. *Cancer Res.*, **47**, 4001.

BAURAIN, R., DEPREZ-DE-CAMPENEERE, D. & TROUET, A. (1979). Determination of daunorubicin, doxorubicin and their fluorescent metabolites by high-pressure liquid chromatography: plasma levels in $DBA_2$ mice. *Cancer Chemother. Pharmacol.*, **2**, 11.

CHIRGWIN, J.M., PRZYBYH, A.C., MACDONALD, R.J. & RUTTER, W.J. (1979). Isolation of biologically active ribonucleic acid from sources enriched in ribonuclease. *Biochemistry*, **18**, 5294.

DANO, K. (1983). Active outward transport of daunomycin in resistant Ehrlich ascites tumor cells. *Biochim. Biophys. Acta*, **323**, 466.

FORMELLI, F., CARSANA, R. & POLLINI, C. (1987). Pharmacokinetics of 4'-deoxy-4'-iodo-doxorubicin in plasma and tissues of tumor-bearing mice compared with doxorubicin. *Cancer Res.*, **47**, 5401.

GANZINA, F. (1983). 4'-Epi-doxorubicin, a new analog of doxorubicin: a preliminary overview of preclinical and clinical data. *Cancer Treat. Rev.*, **10**, 1.

GIANNI, L., SURBONE, A., VIGANO, L., GAMBETTA, A.R. & BONADONNA, G. (1989). Pharmacokinetic guidelines for phase I dose escalations: the case of 4'-dehydroxy-4'-iododoxorubicin. 6th NCI-EORTC Symposium, Amsterdam, March 7–10, 297.

HANAHAN, D. (1983). Studies on transformation of *Escherichi coli* with plasmids. *J. Mol. Biol.*, **166**, 557.

ISRAEL, M., PEGG, W.J., WILKINSON, P.M. & GARNICK, M.B. (1978). Liquid chromatographie analysis of adriamycin and metabolites in biological fluids. *J. Liquid Chromatogr.*, **1**, 795.

LOWRY, O.H., ROSEBROUGH, N.J., FARR, A.L. & RANDALL, R.S. (1951). Protein measurement with the Folin phenol reagent. *J. Biol. Chem.*, **193**, 265.

MANIATIS, T., FRITSCH, E.F. & SAMBROOK, J. (1982). *Molecular Cloning, a Laboratory Manual.* Cold Spring Harbor Laboratory: Cold Spring Harbor, New York.

ROBERT, J., RECONDO, G. ALAKL, M., ORLANDI, F., HURTELOUP, P. & ARMAND, J.P. (1989). Pharmacokinetics and metabolism of 4'-iodo-4'-deoxydoxorubicin in advanced cancer patients. 5th International Conference on Clinical Oncology, London, 3–7 September.

SCHOTT, B. & ROBERT, J. (1989). Comparative cytotoxicity, DNA synthesis inhibition and drug incorporation of eight anthracyclines in a model of doxorubicin-sensitive and resistant rat glioblastoma cells. *Biochem. Pharmacol.*, **38**, 167.

SCHWEITZER, J. & GŒRTTLER, K. (1980). Synthesis in vitro of keratin polypeptides directed by mRNA isolated from newborn and adult mouse epidermis. *Eur. J. Biochem.*, **112**, 243.

SOUTHERN, E.M. (1975). Detection of specific sequences among DNA fragments separated by gel electrophoresis. *J. Mol. Biol.*, **98**, 503.

THOMAS, P. (1980). Hybridization of denatured RNA and small DNA fragments transferred to nitrocellulose. *Proc. Natl Acad. Sci. USA*, **77**, 5201.

TSURUO, T., IIDA-SAITO, H., KAWABATA, H., OH-HARA, T., HAMADA, H. & UTAKOJI, T. (1986). Characteristics of resistance to adriamycin in human myelogenous leukemia K562 resistant to adriamycin and in isolated clones. *Jap. J. Cancer Res.*, **77**, 682.

VAN DER BLIEK, A., VAN DER VELDE-KŒRTS, T., LING, V. & BORST, P. (1986). Overexpression and amplification of five genes in a multidrug resistant chinese hamster ovary cell line. *Mol. Cell. Biol.*, **6**, 1671.

VRIGNAUD, P., LONDOS-GAGLIARDI, D. & ROBERT, J. (1986*a*). Cellular pharmacology of doxorubicin in sensitive and resistant rat glioblastoma cells. *Oncology*, **43**, 60.

VRIGNAUD, P. MONTAUDON, D., LONDOS-GAGLIARDI, D. & ROBERT, J. (1986*b*). Fatty acid composition, transport and metabolism in doxorubicin-sensitive and -resistant rat glioblastoma cells. *Cancer Res.*, **46**, 3258.